# Closing the gap: mixed stock analysis of three foraging populations of green turtles (*Chelonia mydas*) on the Great Barrier Reef

**DOI:** 10.7717/peerj.5651

**Published:** 2018-09-28

**Authors:** Karina Jones, Michael Jensen, Graham Burgess, Johanna Leonhardt, Lynne van Herwerden, Julia Hazel, Mark Hamann, Ian Bell, Ellen Ariel

**Affiliations:** 1 College of Public Health, Medical and Veterinary Sciences, James Cook University of North Queensland, Townsville, Australia; 2 Centre for Sustainable Tropical Fisheries and Aquaculture, James Cook University, Townsville, Queensland, Australia; 3 Centre for Tropical Water and Aquatic Ecosystem Research, James Cook University, Townsville, Queensland, Australia; 4 Marine Mammal and Turtle Division, National Marine Fisheries Service, National Oceanic and Atmospheric Administration, La Jolla, California, CA, USA; 5 ARC Centre of Excellence for Coral Reef Studies, James Cook University, Townsville, Queensland, Australia; 6 Discipline of Marine Biology, James Cook University, Townsville, Queensland, Australia; 7 College of Science and Engineering, James Cook University, Townsville, Queensland, Australia; 8 Department of Environment and Science, Queensland Government, Townsville, Queensland, Australia

**Keywords:** Mixed stock analysis, Marine turtle, Spatial ecology, Genetics, *Chelonia mydas*, Mitochondrial DNA, Great Barrier Reef

## Abstract

A solid understanding of the spatial ecology of green turtles (*Chelonia mydas*) is fundamental to their effective conservation. Yet this species, like many marine migratory species, is challenging to monitor and manage because they utilise a variety of habitats that span wide spatio-temporal scales. To further elucidate the connectivity between green turtle rookeries and foraging populations, we sequenced the mtDNA control region of 278 turtles across three foraging sites from the northern Great Barrier Reef (GBR) spanning more than 330 km: Cockle Bay, Green Island and Low Isles. This was performed with a newly developed assay, which targets a longer fragment of mtDNA than previous studies. We used a mixed stock analysis (MSA), which utilises genetic data to estimate the relative proportion of genetically distinct breeding populations found at a given foraging ground. Haplotype and nucleotide diversity was also assessed. A total of 35 haplotypes were identified across all sites, 13 of which had not been found previously in any rookery. The MSA showed that the northern GBR (nGBR), Coral Sea (CS), southern GBR (sGBR) and New Caledonia (NC) stocks supplied the bulk of the turtles at all three sites, with small contributions from other rookeries in the region. Stock contribution shifted gradually from north to south, although sGBR/CS stock dominated at all three sites. The major change in composition occured between Cockle Bay and Low Isles. Our findings, together with other recent studies in this field, show that stock composition shifts with latitude as a natural progression along a coastal gradient. This phenomenon is likely to be the result of ocean currents influencing both post-hatchling dispersal and subsequent juvenile recruitment to diverse coastal foraging sites.

## Introduction

Migratory marine mega vertebrates are often long lived and utilise a variety of habitats that span wide spatio-temporal scales. Humpback whales (*Megaptera novaeangliae*), for example, utilise distinctly separate feeding and breeding grounds and undergo seasonal migrations between these areas which can span thousands of kilometres (Acevedo et al., 2007; Clapham, 1996; Oña, Garland & Denkinger, 2017). The same is true of various species of sharks, rays, tuna, marine mammals and marine turtles (Lascelles et al., 2014). Species with complex life history patterns pose challenges to the understanding of population dynamics and the connectivity between breeding and non-breeding areas (Godley et al., 2010). Due to their wide-ranging movements, marine migratory species are exposed to different threats at their foraging and breeding habitats, and are further exposed to additional pressures as they migrate between these habitats (Jensen et al., 2016; Lascelles et al., 2014). These species often pass through the waters of multiple nations or areas beyond national jurisdiction (Lascelles et al., 2014) and as a result, monitoring, managing and ultimately conserving such species is challenging (Hamann et al., 2010; Jensen et al., 2016). In 2014, 48% of all marine migratory species were found to be threatened (critically endangered, endangered or vulnerable), near threatened or data deficient, with marine turtles being the most threatened group (Lascelles et al., 2014). A sound understanding of the spatial ecology of these species is essential to developing effective conservation strategies (Cooke, 2008), as it allows for the identification of key habitats and the likely sources of threatening processes.

The green turtle (*Chelonia mydas*) is recognised as endangered under the IUCN red list assessment (Seminoff, 2004). In Australia, this species is listed as vulnerable under the *Environment Protection and Biodiversity Conservation Act 1999* (Department of Environment Energy, 2016). Green turtles have a circumglobal distribution, are long-lived, highly migratory, and have a complex life history which spans a diverse range of habitats (Limpus, 2008). After emerging from tropical and subtropical sandy beaches hatchling green turtles take on a pelagic existence, recruiting into benthic, inshore foraging grounds as juveniles several years later (Reich, Bjorndal & Bolten, 2007). Foraging areas are often shared by turtles sourced from multiple regional rookeries (Anderson, Shaver & Karel, 2013; Dutton et al., 2014; Lahanas et al., 1998). At the onset of sexual maturity, some 20–30 years later, green turtles migrate back to their natal nesting regions to breed and nest (Musick & Limpus, 1997).

Using mtDNA, Australian green turtles can be divided into nine genetically distinct breeding stocks: southern Great Barrier Reef (sGBR), Coral Sea (CS), northern GBR (nGBR), Gulf of Carpentaria, Coburg Peninsula, Ashmore Reefs/Browse Island, Scott Reef, the Northwest Shelf and Cocos “Keeling” Island (Dethmers et al., 2006; Limpus, 2008; FitzSimmons & Limpus, 2014; Jensen et al., 2016). In addition, Australian waters are in close proximity to multiple internationally important stocks in neighbouring countries such as those nesting in Aru (Indonesia), Papua New Guinea and New Caledonia. Each of these stocks can be considered as a demographically independent population (Waldman, 2005), and as such, understanding how turtles from these stocks share regional foraging grounds is critical to the effective management of threats to this vulnerable species.

The Great Barrier Reef (GBR) region in Australia is home to some of the largest nesting and foraging green turtle populations in the world. Breeding green turtles of nGBR and sGBR stocks nest on several islands at the latitudinal extremes of the GBR (Dethmers et al., 2006; FitzSimmons & Limpus, 2014; Jensen et al., 2016). While very little nesting takes place along the central part of the reef, turtles from both breeding stocks share foraging areas located along the entire GBR (Limpus, 2008) and beyond into New South Wales and northern Australia. Foraging grounds along the GBR are discontinuous and irregularly spaced, likely reflecting the patchy nature of resources relevant for turtles. For research and monitoring purposes, GBR foraging grounds are defined by their geographical location, e.g., a bay or a cluster of neighbouring reefs. These foraging grounds typically support overlapping adult and juvenile age classes. Long-term mark-recapture studies have demonstrated that all size classes have strong fidelity to a single foraging ground with little movement between surrounding foraging grounds (Limpus & Chaloupka, 1997; Musick & Limpus, 1997). As such, GBR foraging grounds are considered to host independent foraging populations wherein the genetic composition is mixed.

Both traditional mark-recapture analysis (flipper tagging) and molecular methods (mixed stock analysis; MSA) have been used to describe the distribution of foraging green turtles along the GBR (Jensen et al., 2016; Limpus, 2008). The MSA method uses genetic markers measured in several source populations (rookeries) and a single mixed population (a foraging ground) to estimate the proportional contribution of each source to the mixed population (Bolker et al., 2007). This technique provides an effective tool to assess the connectivity between foraging and breeding grounds for migratory species like marine turtles, whose intricate life history complicates monitoring efforts. Major green turtle rookeries across the Indo-Pacific have been genetically characterised using the mtDNA control region, with 25 genetically differentiated stocks or Management Units (MUs) identified to date ( Dutton et al., 2009; Dutton et al., 2014; Jensen et al., 2016; Nishizawa et al., 2014; Read et al., 2015). These MUs provide a comprehensive reference of source populations that can be used in MSA to determine the breeding stock origin of green turtles at regional foraging grounds along the GBR and elsewhere (Dethmers et al., 2006; FitzSimmons & Limpus, 2014; Jensen et al., 2016; Limpus, 2008).

Studies based on traditional flipper tagging, genetic data, or a combination of these tools have shown that foraging areas along the GBR mainly receive turtles originating from three stocks; the nGBR, the sGBR and the Coral Sea (CS) (Jensen et al., 2016; Limpus, 2008). In addition to these dominant breeding stocks, small proportions of turtles foraging in these locations are supplied by more distant rookeries (Jensen et al., 2016; Limpus, 2008). The composition of stocks at foraging grounds along the GBR also alters with latitude; northern foraging grounds are mostly populated with turtles originating from the nGBR breeding stock whilst sGBR and CS stocks are more prominent in southern foraging grounds (Jensen et al., 2016).

**Figure 1:**
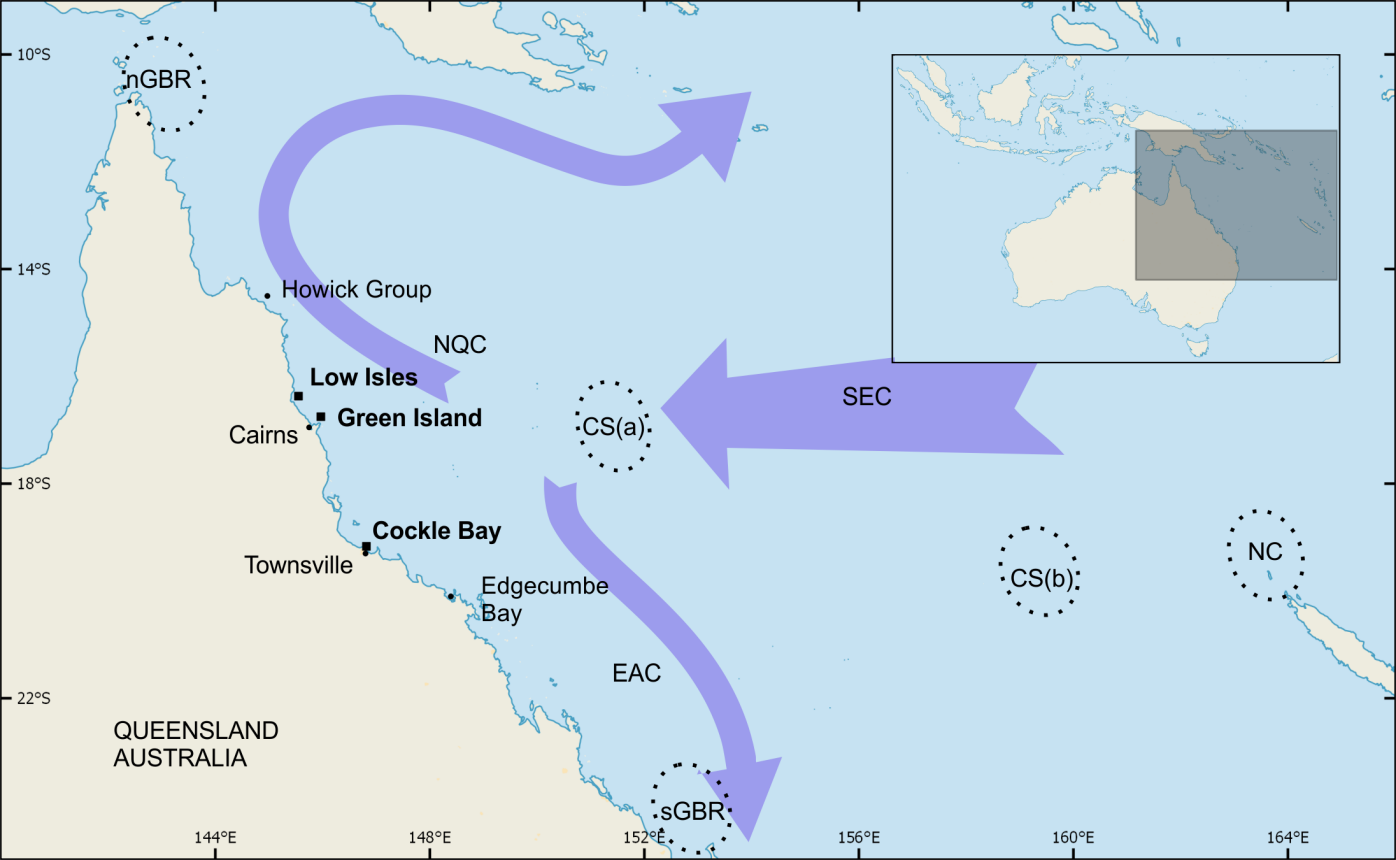
Green turtle foraging sites and genetic stocks of interest to this study. Green turtle foraging sites at Low Isles, Green Island and Cockle Bay were sampled for genetic analysis in the present study. These three sites filled a large geographic gap that existed in prior sampling by Jensen et al. (2016). Broken-line ellipses indicate breeding areas of the following source populations: northern GBR (nGBR), Coral Sea comprised of Coringa-Herald group (CS(a)) and Chesterfield group (CS(b)), southern Great Barrier Reef (sGBR), and New Caledonia (NC). Arrows provide a simplified representation of ocean currents in the region of interest: NQC, North Queensland Current; EAC, East Australian Current; SEC, South Equatorial Current.

This latitudinal variance can be observed on a broad scale (north to south, as above) and also on a finer scale (between specific foraging grounds). A major shift in the stock composition between the more northerly Howick Group of islands and the more southerly Edgecumbe Bay (Fig. 1) has been described using a combination of MSA and flipper-tag returns (Jensen et al., 2016). While foraging turtles at Edgecumbe Bay were predominantly from the sGBR and CS stocks, turtles at the Howick Group were a mixture of sGBR, CS and nGBR stock. However, there was a large geographic gap in the sampling of foraging grounds between the Howick Group and Edgecumbe Bay spanning six degrees of latitude and approximately 700 km. Assessing the stock composition at foraging grounds within this spatial gap would further refine our knowledge of the latitude at which the composition of green turtles shifts from predominantly sGBR to predominantly nGBR turtles. Furthermore, closing this knowledge gap and combining these results with already published data may provide a means of assessing the relationship between stock composition and latitude. If such a relationship exists, it may provide a means to predict stock composition at other un-sampled foraging grounds in this region. Therefore, in this study, we (1) generated and used mtDNA control region sequences and MSA to quantify the stock composition of green turtles at three foraging areas located between Edgecumbe Bay and the Howick Group, and (2) used our new data and data from previously sampled foraging areas to assess the correlation between stock composition and latitude of foraging areas in Eastern Australian waters.

## Materials and Methods

### Study sites

Green turtles were sampled during separate projects at three foraging grounds within the Great Barrier Reef, Queensland, Australia (Fig. 1). The sites listed below are in north to south order.

#### Low Isles (LI)

(16°22′S, 145°33′E), situated 15 km off the mainland of North Queensland, comprises two small islands on a shallow coral reef. Turtles at this site were sampled between June 2010 and November 2011.

#### Green Island (GI)

(16°45′S, 145°58′E), a coral cay located in the northern GBR region approximately 27 km offshore from Cairns, Queensland was sampled in October 2012.

#### Cockle Bay (CB)

(19°10′S, 146°49′E) is a small bay of Magnetic Island, located approximately eight km offshore from Townsville, the largest tropical city in Australia. Sampling of this site was conducted in August and November of 2012.

### Sample collection

Turtles at all three sites were captured by rodeo method (Limpus & Reed, 1985). Captured turtles were flipper-tagged with a unique alpha-numeric inscribed titanium tag (Stockbrands Company, Pty. Ltd., Perth, Western Australia), and had their curved carapace length (CCL ± 1 mm) measured using a flexible tape measure. Skin samples (approx. 5 × 5 mm) were collected using a sterilised scalpel for each turtle. At CB and GI the skin samples were taken from the neck and stored in a 20% DMSO solution saturated with NaCl. At LI the samples were collected from the trailing edge of the front flipper and stored in 90% ethanol. Samples from a total of 278 turtles were collected (see Table 1 for details).

**Table 1:** Sample demographics. Green turtle demographics from three sampled Great Barrier Reef foraging grounds. The total number of turtles sampled per site (*n*), and number of juvenile (J), sub-adult (SA) and adult (A) turtles within each site are shown. Curved-carapace length (CCL) mean and range are also provided.

**Foraging ground**	*n*	**Size class**	**Mean CCL (cm)**	**Range of CCL (cm)**
Low Isles (LI)	147	114 J; 33 SA; 0A	55.2	39.7–80.2
Green Island (GI)	57	52 J; 5 SA; 0A	50.4	47.0–84.4
Cockle Bay (CB)	74	58 J; 12 SA; 4A	54.5	40.2–103.9

Sample collection at Cockle Bay and Green Island was conducted under scientific research permit G12/35326.1 by an appointed conservation officer under the Nature Conservation Act 1992 during population monitoring. Sample collection at Low Isles was conducted under James Cook University Ethics Approval A1474 and scientific research permits G10/33206.1, G10/33897.1 & WISP06563509.

### DNA extraction and Polymerase Chain Reaction (PCR)

#### Cockle Bay and Green Island

DNA from the CB and GI samples was extracted using the Promega Wizard^®^ SV Genomic DNA Purification System (Promega, Madison, WI, USA) according to the manufacturer’s instructions. An extra 10 µL of proteinase K was used per reaction. Final DNA concentration was obtained by spectrophotometric analysis, using the ratios of absorption at 260 nm versus 280 nm to determine DNA purity.

The primers ChM-Dloop-960 F (5′-AAC TAT AAC CTT CCT AGA-3′) and ChM-Dloop-960 R (5′-TGT AAG TAT CCT ATT GAT T-3′) were designed to target a 960 bp region of the mtDNA d-loop control region in green turtles. These primers were designed in AlleleID v7 using an alignment of 15 published green turtle sequences. These primers were optimised in conventional polymerase chain reaction (PCR) using a gradient of 50 °C–60 °C.

PCRs were carried out in 20 µL reactions consisting of 10 µL GoTaq Green Hot Start Master Mix (Promega, Madison, WI, USA), 0.8 µM of each primer, ∼80 ng of template DNA and nuclease free water to 20 µL.

The PCR protocol consisted of a 5 min denaturation step (94 °C) followed by 35 cycles of: 10 s at 94 °C, 15 s at 54 °C, and 30 s at 72 °C and a final extension step of 5 min at 72 °C. PCR products were visualised on a 1.2% agarose gel. Following assay optimisation, PCR products were visualised in real time using 20 µL reactions consisting of 10 µL GoTaq qPCR Master Mix (Promega, Madison, WI, USA), 0.8 µM of each primer, ∼80 ng of template DNA and nuclease free water to 20 µL. The qPCR protocol consisted of a 2 min denaturation step (95 °C) followed by 45 cycles of: 10 s at 95 °C, 30 s at 51 °C, and 30 s at 72 °C. These products were then sent to Macrogen (Macrogen Inc., Seoul, Korea) for purification and sequencing using both the forward and reverse primers to initiate sequencing. A consensus sequence was subsequently generated and used in further analysis.

#### Low Isles

The DNA extraction from LI samples was performed using a salting out procedure, based upon Sunnucks & Hales (1996). Genomic DNA concentration and quality of the LI samples was evaluated through gel electrophoresis in the presence of GelGreen (Biotium, Fremont, CA, USA).

Partial mtDNA d-loop control region (760 bp) was amplified using the primers LTEi9 (5′GAATAATCAAAAGAGAAGG 3′) and H950 (5′GTCTCGGATTTAGGGGTTT 3′) (Abreu-Grobois et al., 2006). PCR was performed in a 25 µL reaction containing 1 × NH_4_ Buffer, 1.5 mM MgCl, 0.25 mM dNTPs, 0.4 µM of each primer, 1 Unit of BioTaqTM polymerase and ∼10ng DNA. The PCR protocol consisted of an initial denaturation step at 94 °C for 5 min, followed by 35 cycles of 45 s at 94 °C, 45 s at 52 °C, and 1 min at 72 °C and a final extension step of 5 min at 72 °C. PCR samples were purified and sequenced by Macrogen (Macrogen, Inc., Seoul, Korea) using ABI Dye terminator chemistry on an ABI 3730 sequencer.

### Characterisation of mtDNA haplotypes and mixed stock analysis (MSA)

All sequences obtained were assembled in Geneious v7.1.5 (Kearse et al., 2012) and confirmed to be the correct target using the database of the Basic Local Alignment Search Tool (BLAST) (https://blast.ncbi.nlm.nih.gov/Blast.cgi). Sequences were trimmed to ∼770 bp to allow comparison with known green turtle haplotypes in the published literature.

These sequences were then compared with known haplotypes and assigned existing names accordingly. Any sequences from three or fewer turtles which did not match any known haplotypes were re-sequenced to a total of three replicates, in order to avoid sequencing error. Where possible, new template DNA was generated from the original sample. Once confirmed, these new haplotypes were named following the nomenclature for Pacific green turtles using the prefix CmP (Jensen et al., 2016).

Haplotype frequency at each site was recorded and haplotype (*h*) and nucleotide diversity (*π*) (Nei, 1987) were estimated using Arlequin version 3.5.2.2 (Excoffier, Laval & Schneider, 2005).

To estimate the proportional contributions of stocks to the three foraging areas, MSA was conducted using a Bayesian approach in the software program Bayes (Pella & Masuda, 2001). The mtDNA haplotype frequencies of 25 genetically distinct green turtle breeding stocks across the Indo-Pacific (see Table S1 in Jensen et al. (2016)) were used as a baseline. As MSA estimates the proportional contributions of stocks to one feeding ground at a time, each study site was analysed independently. Each analysis consisted of 4 independent chains with different starting points. Each chain was run for a total of 50,000 steps discarding the first 25,000 steps as burn-in. To determine whether all chains had converged we used the Gelman and Rubin shrink factor diagnostic (shrink factor <1.2) (Pella & Masuda, 2001). The analysis was conducted with both uniform priors (Model 1) and weighted priors (Model 2). In Model 2 the priors were weighted according to the nesting population size associated with each stock. The results were summarised for both individual stocks and regional estimates grouping the sGBR and CS (sGBR/CS) as well as 21 stocks that all contributed <5% (other).

## Results

The sequence data of a 770 bp fragment of the d-loop control region was obtained from 278 individual turtles across three foraging sites. A total of 35 haplotypes were identified, 13 of which had never been observed at a rookery (orphan haplotypes). Eight of this subset were previously undescribed; one at Cockle Bay (CmP80.4), four at Green Island (CmP234.1, CmP235.1, CmP236.1 and CmP237.1), and three at Low Isles (CmP145.1, 166.2 and CMP211.1). The remaining five haplotypes had been described in previous studies, but had also not yet been observed at a rookery (CmP34.1, CmP55.1, CmP119.1, CmP165.1 and CmP200.1) (Table 2). These orphan haplotypes occurred at low frequencies (2.7–10.5%) and comprised only 6.5% of the total number of turtles sampled. The most common haplotype observed was CmP47.1 at all three sites; CB (73%), GI (67%) and LI (53%) (Table 2). Haplotype and nucleotide diversity both increased from south to north along the GBR, although CB and GI share similar nucleotide diversity (Table 3).

### Mixed stock analysis

The MSA showed that the nGBR, CS, sGBR and New Caledonia (NC) stocks supplied the bulk of the turtles at all three sites (>91.6% overall) (Table 4). Small contributions were also made by other more distant green turtle rookeries in the region, but together they made up ∼8% at each site. Both Model 1 (uniform priors) and Model 2 (weighted priors) yielded similar results (Table 4), and for the purpose of simplicity, we only discuss results from Model 2 from hereon. Given the uncertainty surrounding small contribution estimates we grouped rookeries with <5% estimated mean contribution into ‘Other’. We were unable to run the MSA for individual age classes due to insufficient sample sizes.

The contribution of NC stocks was approximately equal at all three sites (Table 2), and in all cases was above that which would be considered a small contribution. However, the nGBR, CS and sGBR stock contributions shifted between sites. Turtles at CB, the most southerly site, predominantly originated from sGBR stocks (82.8%, 95% CI [68.9–92.9]), with small contributions from nGBR (8.0%, 95% CI [2.0–16.4]) and CS (0.6%, 95% CI [0.0–5.4]) stocks, respectively. The CS stock was dominant at both GI and LI (approximately 50% to 60%, respectively). As a general trend, the contributions of nGBR stock increased from south to north, whilst the sGBR stock contributions simultaneously decreased. The most dramatic shifts in nGBR stock contributions were observed between GI and LI; nGBR contributions increased from 3.7% (95% CI [0.0–13.3]) at GI to 15.0% (95% CI [8.9–22.3]) at LI and the sGBR contributions decreased from 38.4% (95% CI [0.0–90.5]) at GI to 11.8% (95% CI [0.0–52.0]) at LI. Interestingly, nGBR stock contributions were lower at GI than CB, despite GI being situated more northerly.

The results also indicate a shift in CS stock contributions from CB to GI, which are separated by approximately 280 km. While the CS contribution is low at CB (0.6%), it makes up the majority of turtles at GI (48.6%, 95% CI [0.0–95.0]) and LI (60.0%, 95% CI [19.7–79.0]). In comparison, the contribution of sGBR is highest at CB (82.8%, 95% CI [68.9–92.9]), medium at GI (38.4%, 95% CI [0.0–90.5]) and lowest at LI (11.8%, 95% CI [0.0–52.0]) (Table 4).

**Table 2:** Haplotype frequencies. Haplotype frequencies of green turtles sampled at Cockle Bay, Green Island and Low Isles along the Great Barrier Reef, Australia.

**Haplotype name**	**Accession number**	**Reference**	**Location**		
			**Cockle Bay (CB)**	**Green Island (GI)**	**Low Isles (LI)**
CmP20.1	AB819806	Hamabata, Kamezaki & Hikida (2014)	–	2	1
CmP20.2	KF311744	Dutton et al. (2014)	–	–	1
CmP22.1	KF311747	Dutton et al. (2014)	1	–	1
CmP40.1	KF311750	Dutton et al. (2014)	–	–	2
CmP44.1	KF311751	Dutton et al. (2014)	4	3	10
CmP44.2	KF311752	Dutton et al. (2014)	–	–	1
CmP47.1	KF311753	Dutton et al. (2014)	54	38	78
CmP49.1	AB819808	Hamabata, Kamezaki & Hikida (2014)	1	–	–
CmP57.2	KJ502567	Jensen et al. (2016)	–	–	3
CmP65.1	KF311756	Dutton et al. (2014)	–	–	1
CmP68.1	KJ502591	Jensen et al. (2016)	–	–	1
CmP77.1	KF311759	Dutton et al. (2014)	–	–	1
CmP80.1	KF311760	Dutton et al. (2014)	8	6	19
CmP81.1	KJ502610	Jensen et al. (2016)	–	–	2
CmP84.1	KJ502630	Jensen et al. (2016)	1	–	1
CmP85.1	KF311761	Dutton et al. (2014)	2	1	3
CmP91.1	KF311762	Dutton et al. (2014)	–	–	2
CmP98.1	FJ917199	Dutton et al. (2009)	–	–	6
CmP168.1	KJ502617	Jensen et al. (2016)	–	–	1
CmP169.1	KJ502608	Jensen et al. (2016)	1	–	–
CmP180.1	KJ502640	Jensen et al. (2016)		–	2
CmP193.1	KJ502635	Jensen et al. (2016)	–	1	1
		**Total**	72	51	137
**Orphan Haplotypes**					
CmP34.1	KJ502581	Jensen et al. (2016)	–	1	–
CmP55.1	KJ502596	Jensen et al. (2016)	–	1	4
CmP80.4	MH004276	This study	1	–	–
CmP119.1	KJ502611	Jensen et al. (2016)	–	–	1
CmP145.1	MH004277	This study	–	–	1
CmP165.1	KJ502582	Jensen et al. (2016)	1	–	–
CmP166.2	MH004278	This study	–	–	2
CmP200.1	KJ502586	Jensen et al. (2016)	–	–	1
CmP211.1	MH004283	This study	–	–	1
CmP234.1	MH004279	This study	–	1	–
CmP235.1	MH004280	This study	–	1	–
CmP236.1	MH004281	This study	–	1	–
CmP237.1	MH004282	This study	–	1	–
		**Total**	2	6	10
		**Cumulative total**	**74**	**57**	**147**

**Table 3:** Haplpotype and nucleotide diversity. Sample size (*n*), number of haplotypes (*H*) and estimates (± SD) of haplotype (*h*) and nucleotide (*π*) diversity for three *C. mydas* foraging sites on the Great Barrier Reef, Australia.

**Foraging site**	*n*	*H*	*h*	*π*
Cockle Bay	74	10	0.4572 ± 0.0694	0.013573 ± 0.006930
Green Island	57	12	0.5476 ± 0.0772	0.012378 ± 0.006384
Low Isles	147	26	0.6970 ± 0.0396	0.019210 ± 0.009563

**Table 4:** Mixed stock analysis results of 278 turtles from three foraging grounds along the Great Barrier Reef. Results (mean% ± 95% confidence intervals in parentheses) from the Bayesian mixed stock analysis (MSA) (Pella & Masuda, 2001) for Cockle Bay, Green Island and Low Isles Green Turtles (both individually and by region). MSA was calculated using 25 regional breeding stocks as possible sources, but for simplicity only the four main contributors are listed—nGBR, northern Great Barrier Reef; sGBR, southern Great Barrier Reef; CS, Coral Sea and NC, New Caledonia. The combined contributions of the remaining 21 stocks are compiled into the ‘Other’ category. Model 1, uniform priors; Model 2, weighted priors.

		**Cockle Bay**		**Green Island**		**Low Isles**	
	**Stock**	**Model 1**	**Model 2**	**Model 1**	**Model 2**	**Model 1**	**Model 2**
**Individual**	**nGBR**	7.5 (1.7–15.8)	8.0 (2.0–16.4)	1.5 (0.0–10.1)	3.7 (0.0–13.3)	14.7 (8.7–21.7)	15.0 (8.9–22.3)
	**CS**	2.5 (0.0–29.4)	0.6 (0.0–5.4)	50.4 (0.9–92.8)	48.6 (0.0–95.0)	60.1 (20.3–78.1)	60.0 (19.7–79.0)
	**sGBR**	79.7 (53.8–91.3)	82.8 (68.9–92.9)	33.7 (0.0–85.2)	38.4 (0.0–90.5)	10.8 (0.0–50.4)	11.8 (0.0–52.0)
	**NC**	7.3 (0.0–19.4)	6.9 (0.0–19.8)	9.4 (0.0–23.6)	6.1 (0.0–22.0)	6.0 (1.5–13.2)	6.3 (1.5–13.7)
	**Other**	3 (0.1–8.7)	1.7 (0.0–6.5)	5.0 (0.3–12.8)	3.2 (0.0–11.6)	8.4 (3.9–14.0)	6.9 (2.8–12.3)
**Regional**	**nGBR**	7.5 (1.7–15.8)	8.0 (2.0–16.4)	1.5 (0.0–10.1)	3.7 (0.0–13.3)	14.7 (8.7–21.7)	15.0 (8.9–22.3)
	**sGBR/CS**	82.2 (70.1–91.7)	83.3 (70.8–93.0)	84.1 (70.2–94.3)	87.0 (73.2–96.4)	70.9 (61.8–79.1)	71.8 (62.6–80.0)
	**NC**	7.3(0.0–19.4)	6.9 (0.0–19.8)	9.4 (0.0–23.6)	6.1 (0.0–22.0)	6.0 (1.5–13.2)	6.3 (1.5–13.7)
	**Other**	3.0 (0.1–8.6)	1.9 (0.0–6.5)	5.1 (0.4–12.9)	3.1 (0.0–11.6)	8.4 (4.0–14.0)	6.9 (2.8–12.3)

**Figure 2:**
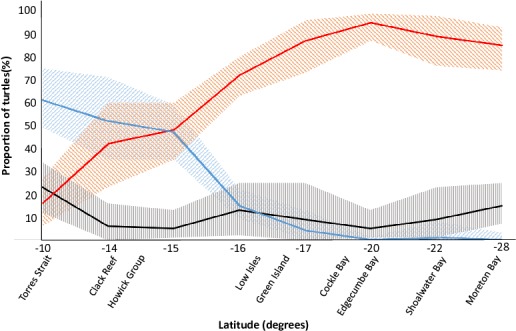
The latitudinal spread of the main genetic stocks on the Great Barrier Reef. For green turtle aggregations at selected foraging grounds in the Great Barrier Reef (GBR) and southern Queensland, Australia, the proportional contributions of three important genetic sources showed a notable relationship with latitude. The southern GBR (sGBR) and Coral Sea (CS) stocks were combined for this figure to allow comparison with Jensen et al. (2016) and are denoted in orange. The nGBR stock (northern GBR) is represented in blue and ‘Other’ stocks, represented in black; hatched areas in all three cases represented 95% confidence intervals. The ‘Other’ group comprises the remaining 22 stocks in this region (see Jensen et al., 2016) and were combined because these stocks were found to contribute a small proportion of the turtles at each study site. Data for Low Isles, Green Island and Cockle Bay from the present study and data for all other sites are from Jensen et al. (2016).

These results, combined with previously published reports, were plotted on a chart which shows the stock composition shifting along a latitudinal gradient (Fig. 2). It is possible that this data could be used to predict stock compositions at sites along this gradient that have not been previously sampled.

## Discussion

Previous studies indicated that foraging grounds along the GBR are dominated by the nGBR, sGBR and Coral Sea genetic stocks and that the proportions of those stocks change gradually from north to south (Dethmers et al., 2006; Jensen et al., 2016). However, a 700 km unsampled gap separated foraging grounds of predominantly nGBR stocks (the Howick Group) and foraging grounds further south where only a small proportion of nGBR turtles were observed (Edgecumbe Bay) (Jensen et al., 2016) (Fig. 1). This sampling gap precluded informed management regarding the stocks that might be impacted at foraging grounds along the central part of the GBR and was therefore the focal area of our study.

Due to the high degree of genetic similarity between the CS and sGBR stocks, the MSA estimates for these stocks are surrounded by high uncertainty. In order to address this, we combined the summary statistics of these genetically similar stocks. However, Read et al. (2015), who also utilised MSA to study turtles in the Indo-pacific region, reported summary statistics for individual stocks. To make our results comparable with both the Jensen et al. (2016) and Read et al. (2015) studies, we present the summary statistics for both individual stocks, as well as the combined CS/sGBR stock (Table 4).

Our results show that gradual changes in stock contribution occur between CB and LI. The combined sGBR/CS stock foraging at CB and GI made up a smaller proportion (83–87%) at these sites compared to the proportion observed at the more southerly Edgecumbe Bay (95%) (Jensen et al., 2016). This proportion decreased further at the more northern LI (72%). The contrary was evident for the nGBR stock that declined from making up half of the juvenile turtles foraging at the Howick group (Jensen et al., 2016) to 15% at LI and decreasing further at GI and CB (4% and 8%, respectively) to 0% at Edgecumbe Bay. In addition, we found that all three study sites (LI, GI and CB) were comprised of a small portion (6–7%) of turtles from the New Caledonia stock, which is derived from rookeries more than 1800 km away. These findings are consistent with both tag-recovery data and MSA results from other studies, suggesting that New Caledonia turtles use multiple feeding grounds along the Great Barrier Reef (Read et al., 2014; Read et al., 2015; Jensen et al., 2016). Interestingly, whilst CS stock was found to contribute a large proportion of the turtles at our study sites, this stock was found to contribute only a small proportion of turtles foraging in New Caledonia (Read et al., 2015).

The shift in the composition of regional stocks at foraging areas along the Great Barrier Reef may in part be explained by ocean currents, as has been suggested for mixed stocks of marine turtles in in other regions (Blumenthal et al., 2009; Carreras et al., 2006; Lahanas et al., 1998; Luke et al., 2004). The three foraging grounds sampled in our study (LI, GI and CB) are geographically situated near an area of variable currents (Choukroun et al., 2010) associated with the South Equatorial Current dividing into the south-flowing East Australian Current and the north flowing North Queensland Current (Fig. 1). Such a split is likely to influence the dispersal of new recruits approaching the Australian east coast following their oceanic phase as they move towards their neritic foraging areas. The high proportion of CS stock observed at our study sites on the GBR compared to the low proportion of this stock observed at New Caledonia (Read et al., 2015) further supports this theory. However, the mechanisms of how these new recruits settle at neritic foraging areas are not known and would be a worthy avenue for future research.

While the vast majority of sampled turtles came from rookeries within the GBR region, Coral Sea and New Caledonia, a small proportion of turtles came from more distant rookeries. The latter stocks were grouped collectively into the ‘Other’ category. However, the distribution of specific haplotypes at regional rookeries reveal their likely origin. For example, CmP20.1 is common throughout Micronesia, CmP22.1 in the Marshall Islands and CmP65.1 has only been found in American Samoa and French Polynesia (see Dutton et al., 2014; Hamabata, Kamezaki & Hikida, 2014). In this study, these haplotypes were infrequently found; two turtles at GI and one turtle at LI were found to be CmP20.1, while CmP22.1 was found once at both CB and LI. One turtle at CB was found to be CmP65.1, making this the first known record of this haplotype on the GBR. These rare long-distance dispersal events are supported by tag returns from turtles as far as the Marshall Islands foraging along the GBR (Limpus, Bell & Miller, 2009).

We identified 13 orphan haplotypes across all three sites and encountered them more frequently in the more northerly sites. These haplotypes were distributed as CB:2, GI:6 and LI:6, with one orphan haplotype (CmP55.1) present at both GI and LI. Eight of these haplotypes were previously undescribed whilst five others had been described in previous studies, but had not yet been observed at a rookery (Jensen et al., 2016). Orphan haplotypes at GI were found to comprise nearly 11% of all turtles sampled. These orphan haplotypes indicate that some of the known rookeries may require larger sample sizes to accurately capture the haplotype composition. It is also possible our study sites may have received turtles from unidentified and unsampled rookeries that might exist in south-east Asia or the south-western Pacific. While these orphan haplotypes highlight the need for additional sampling of green turtle rookeries in the region, it is encouraging that they only comprise a small percentage (<6.5%) of our total data set.

The Chm-dloop 960 primer set described here is specific to green turtles and can be used to obtain a longer (960 bp) fragment of the d-loop control region, thereby allowing for an improved resolution. Many of the haplotypes in this study are shared between a number of stocks (e.g., CmP80.1 is found in the nGBR, sGBR, Coral Sea and New Caledonia stocks). However, when analysing the longer fragment of mtDNA, this haplotype could be consistently split into two distinct haplotypes and potentially add resolution to the stock structure of those populations. Therefore, future studies may benefit from using this assay, or preferably designing primers that target the entire d-loop region. In particular, this increased resolution may aid in resolving any uncertainty in separating the sGBR and CS stocks in the MSA. Moreover, such work may allow researchers to more reliably distinguish the region of origin for particular haplotypes (for example, tracing a certain haplotype back to one stock instead of four).

As marine turtles have a complex life history, it is important that conservation strategies target the full range of life stages and habitats used by these turtles. In order to effectively manage threats to green turtles, we must understand the size of the stocks and the factors that are threatening them (Hamann et al., 2010). The identification of individual green turtle stocks present on the GBR has greatly improved the monitoring and management of this species by allowing a more targeted approach. Each stock is considered to be a separate management unit that is demographically independent, hence a decline in one stock would not be replenished by another (Dobbs, 2001; Waldman, 2005). As a result of unsustainable commercial harvesting of green turtles in the southern GBR in the early to mid 1900s (Limpus, 2008), the sGBR stock presumably declined. While the sGBR populations are presently recovering (Chaloupka et al., 2008; Department of Environment Energy, 2016; GBRMPA, 2014; Limpus, 2008), the pressure from historical consumptive use may have affected the distribution of this stock or the composition of different age classes. Similarly, the nGBR stock has demonstrated a plateau and there is the potential for a decline in population size due to decreased hatchling success at Raine Island (Chaloupka et al., 2008; GBRMPA, 2014; Limpus et al., 2003). This may already be reflected in the results from the present study, and it is likely that the nGBR contributions to these foraging grounds will decrease further in the future, increasing the urgency for effective conservation strategies which target threats to this stock. Our work confirms that threats to green turtles which occur in GBR foraging areas north and south of Low Isles will predominantly affect the nGBR stock and sGBR/CS stocks respectively. In the present study, we also show that the CS stock likely contributes significant proportions of turtles at both LI and GI with approximately half of the GI green turtles identified as CS stock. This alone indicates that in order to effectively protect green turtles residing in this region of the GBR, we must extend monitoring and conservation efforts to include the CS rookeries because there are currently no monitoring data available for the Coral Sea, making it difficult to know the status of this stock.

## Conclusions

The GBR supports a large number of foraging marine turtles, yet monitoring has only occurred at a small number of sites because monitoring programs (and associated studies such as ours) in this region are often logistically challenging to establish and maintain, requiring both considerable funding and uniquely-skilled persons. Our data provides confirmation and improved resolution to show the current latitudinal spread of haplotypes of turtles inhabiting the GBR. Turtles at foraging sites north of the Howick Group are more likely to originate from the nGBR stock while turtles foraging south of LI appear more likely to come from CS and/or sGBR stock. These distribution patterns could potentially be influenced by declines or increases in nesting success at the major rookeries in the future and should therefore be regarded as a representation of the current situation. However, the steady shift in stock composition highlighted in this paper (Fig. 2) may provide a means to predict the stock composition at other un-sampled foraging grounds in this region in order to make more informed management decisions while circumventing the need to sample and assess additional locations. Continued monitoring of these stocks will allow managers to develop targeted management plans and effectively conserve this iconic species.

## Additional Information and Declarations

### Field Study Permissions

The following information was supplied relating to field study approvals (i.e., approving body and any reference numbers): Sample collection at Cockle Bay and Green Island was conducted under scientific research permit G12/35326.1 by an appointed conservation officer under the Nature Conservation Act during population monitoring. Sample collection at Low Isles was conducted under scientific research permits G10/33206.1, G10/33897.1 & WISP06563509.

### Data Availability

The following information was supplied regarding data availability: The newly described haplotypes were available at GenBank and all accession numbers have been listed in Table 2 (MH004276, MH004277, MH004278, MH004283, MH004279, MH004280, MH004281 and MH004282).
